# The first 1000 days of life: traffic-related air pollution and development of wheezing and asthma in childhood. A systematic review of birth cohort studies

**DOI:** 10.1186/s12940-021-00728-9

**Published:** 2021-04-17

**Authors:** Alessandra Bettiol, Elena Gelain, Erika Milanesio, Federica Asta, Franca Rusconi

**Affiliations:** 1 Department of Experimental and Clinical Medicine, University of Florence, Florence, Italy; 2 Coordinating Centre for Paediatric Rare Diseases, Meyer Children’s University Hospital, Florence, Italy; 3 Azienda Sanitaria Locale (ASL) TO 4, Ivrea (Turin), Italy; 4 Department of Epidemiology, Lazio Regional Health Service, ASL Roma 1, Rome, Italy; 5 Unit of Epidemiology, Meyer Children’s University Hospital, Viale Pieraccini 24, 50139 Florence, Italy

**Keywords:** Air pollution, Asthma, Children, Cohort studies, Early life, Pregnancy, Wheezing

## Abstract

**Background:**

The first 1000 days of life -including pregnancy and the first 2 years after birth- represent a critical window for health interventions.

This systematic review aimed to summarize the evidence on the relationship between traffic-related air pollutants exposure in the first 1000 days of life and the development of wheezing and asthma, with a particular focus on windows of exposure.

**Methods:**

Medline and Embase were searched from January 2000 to May 2020 to retrieve population-based birth-cohort studies, including registries, providing quantitative information on the association between exposure to traffic-related air pollutants during pregnancy or early life, and the risk of developing wheezing and asthma in childhood. Screening and selection of the articles were completed independently by three reviewers. The quality of studies was assessed using the Newcastle-Ottawa scale.

**Results:**

Out of 9681 records retrieved, 26 studies from 21 cohorts were included. The most common traffic-related air pollutant markers were particulate matter (PM) and nitric oxides (NOx). The variability in terms of pollutants, exposure assessment methods, and exposure levels chosen to present the results did not allow a meta-analysis. Exposure to PM and NOx in pregnancy (10 cohorts) was consistently associated with an increased risk of asthma development, while the association with wheezing development was unclear. The second trimester of pregnancy seemed to be particularly critical for asthma risk. As for exposure during early life (15 cohorts), most studies found a positive association between PM (7/10 studies) and NOx (11/13 studies) and the risk of asthma development, while the risk of wheezing development was controversial. The period of postnatal exposure, however, was less precisely defined and a partial overlap between the period of exposure measurement and that of outcome development was present in a consistent number of studies (14 out of 15) raising doubts on the associations found.

**Conclusions:**

Traffic-related air pollution during pregnancy is associated with an increased risk of asthma development among children and adolescents. The relationship between exposure in the first two years of life and the development of wheezing and asthma needs to be confirmed in studies with more precise exposure assessment.

**Supplementary Information:**

The online version contains supplementary material available at 10.1186/s12940-021-00728-9.

## Background

The period from conception to the child’s second year of life (the first 1000 days) is a window for intervention to improve child and adult health [1]. This has been suggested for different exposures and outcomes, especially in the field of nutrition, cognitive development, and respiratory health [2, 3]. Several programmes have therefore been undertaken worldwide with the aim of promoting early life interventions for children and families [1, 4].

Among early risk factors critical for respiratory health, tobacco smoke exposure, especially during pregnancy and in the first months after birth, is well known to be associated with an abnormal lung development and with an increased risk of both wheezing and asthma in offspring [5, 6]. In fact, although lung growth occurs from conception to early adulthood, prenatal and early postnatal periods might be particularly vulnerable time windows [7].

Tobacco smoke and air pollution exposures are not equivalent, but air pollution exposure might have similar consequences for the lungs [7]. The advent of new technologies with a detailed assessment of exposure to air pollutants and a more precise spatial resolution allows nowadays to better explore the association between exposure to air pollutants from conception through infancy and respiratory outcomes later in life. Prospective birth cohorts represent the best design to assess the temporal relationship between early life exposures and the onset of respiratory diseases in childhood.

To date only one systematic review considering birth cohort studies published until March 2014 has focused on the relationship between childhood traffic-related air pollution exposure and subsequent asthma, wheeze, and allergic diseases [8]. Among the 11 cohort studies included in this systematic review [8], eight were population-based, while three were high-risk cohorts (i.e. including only subjects with a family history of asthma or allergies). Furthermore, almost all studies evaluated postnatal exposure, as studies on pregnancy exposure have been published later.

Since 2014, several birth cohort studies have focused on the association between exposure to traffic-related air pollutants, including gases - in particular nitrogen oxides (NO_X_)- and particulate matter (PM) in pregnancy and in the first 2 years after birth and development of respiratory problems in childhood, namely wheezing and asthma.

On these bases, we aimed to systematically review the evidence from population-based birth cohort studies on the relationship between traffic-related air pollutants exposure in utero and in the first 2 years after birth (the first 1000 days of life) and the subsequent development of wheezing and asthma in childhood, with a particular focus on the critical time windows of exposure. A precise identification of the more vulnerable periods of exposure would be important to choose more efficacious preventive measures.

## Methods

We searched Medline and Embase for papers published in English between January 1st 2000 and May 5th 2020.

We considered as eligible only prospective unselected pregnancy or birth cohort studies, including population-based registries, providing quantitative information on the association between exposure to traffic-related air pollutants during pregnancy or during the first 2 years of infant’s life, and the risk of developing wheezing and/or asthma in children and adolescents (aged 1 to 17 years). Cohorts of susceptible populations, such as offspring of parents with asthma and/or allergies, were excluded. We considered exposures to any established traffic-related air pollutant, including black carbon (BC), carbon monoxide (CO), elemental carbon (EC), NOx, nitric oxide (NO), nitrogen dioxide (NO_2_), hydrocarbons, and PM such as Ultra-Fine Particles ≤0.1 μm in diameter (UFPs), PM < 2.5 and < 10 μm in diameter (PM_2.5_, PM_10_), PM between 2.5 and 10 μm in diameter (PM coarse), and soot (i.e., black substance formed by combustion or separated from fuel during combustion, rising in fine particles). We excluded studies that: a) were reviews, commentaries, governmental reports, letters, animal and experimental studies; b) only examined adulthood asthma; c) only examined non-traffic-related air pollutants including ozone (O_3_) which is not emitted directly from automobiles, sulphur dioxide (SO_2_), indoor air pollution, proximity to point sources and wood smoke; d) only examined the association between the exposure to the selected pollutants and asthma exacerbations or severity; e) did not report the estimates of the quantitative association between traffic-related air pollutants and wheezing or asthma development.

The strategies used for Medline and Embase literature search are reported in supplementary Table 1. Briefly, search terms related to the three main thematic areas “traffic-related air pollutants”, “wheezing/asthma” and “paediatric population” were combined through the Boolean operator “AND”.

Titles and abstracts of all records retrieved by the search were screened by three co-authors (AB, EG, EM). We retrieved the full-text and supplementary material of all articles initially identified for potential inclusion. All potentially relevant full texts were independently screened by two pairs of co-authors to check the fulfilment of the inclusion criteria. Discrepancies were resolved through discussion.

In addition, we checked the reference list of previous published systematic reviews on this topic, to identify additional original research papers not retrieved by our search.To avoid study duplication, the following rules were adopted: a) where multiple publications were based on the same birth cohort or registry and considered the same exposures and outcomes within the same children’s age group, only the most recent publication was included; b) where multiple publications were based on the same birth cohort or registry and evaluated the same exposures and respiratory outcomes for different age groups, we selected the publication with the earliest period of wheezing assessment, and the latest period of asthma assessment. The rationale for this choice was that wheezing occurring in the first years of life could have a different meaning in terms of prognosis with respect to wheezing and asthma at older ages and that asthma can be hardly diagnosed in the earlies years of life.

Data were extracted using a standardized form. Two authors (AB, EG) independently extracted the following data:

1. Exposure data: traffic-related air pollutants studied; mean or median or interquartile range (IQR) concentrations; period of exposure; method for exposure assessment.
2. Outcome data: outcome definition; method used to assess the outcome; period of outcome assessment; relevant adjusted effect estimates and 95% Confidence Intervals (CI).
3. Other information: study population; year of publication; sample size; country in which participants were recruited.

The methodological quality of the studies was assessed by two authors (EM and AB) using the Newcastle-Ottawa Quality Assessment Scale for cohort studies [9].

## Results

**Fig. 1:**
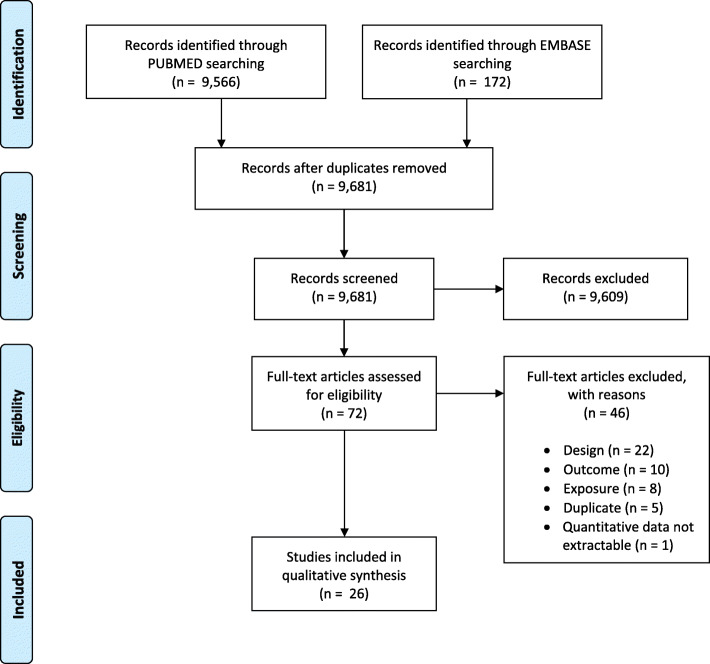
Literature search strategy and results

Our search yielded to 9738 records. After removing duplicates, 9681 unique articles were identified. Of them, 9609 records were excluded after title and abstract screening, whereas 72 articles were selected for full-text reviewing. Among these, 26 articles [10–35] fulfilled the inclusion criteria (Fig. 1).

The 26 articles included in the review were based on 21 pregnancy or birth cohorts.

Nine birth cohorts were registry-based [14–17, 20, 21, 27, 28, 34, 35]. Two studies were case-control study, nested in a registry-based birth cohort [21, 35].

Ten cohorts were based in Europe [11, 13, 22–26, 29–34] and eight were based in North America [14, 16–21, 27, 28, 35]. Of the remaining three cohorts, two were based in Asiatic countries [10, 15] and one in Mexico [12]. Only four birth cohorts reported exposure to air pollutants both in pregnancy and in the first 2 years after delivery [13, 15–17].

The association between exposure to traffic-related air pollutants during pregnancy and the first 2 years of the child’s life and subsequent asthma was evaluated in six [14–21] and 13 cohorts [15–17, 22, 23, 27–35], respectively (Tables 1 and 2**).**

**Table 1:** Association between exposure to traffic related air pollutants in pregnancy and wheezing development

References	Type of study, country	Subjects, no	Pollutants and exposure assessment	Outcome	Positive association with the outcome	Sensitivity windows
Soh S et al., 2018 [10]	GUSTO birth cohort, Singapore	953	PM_2.5_ Daily exposure	Wheezing (birth up to 2 years)	Yes	No sensitive trimester
Madsen C et al., 2017 [11]	MoBa, pregnancy cohort, Norway	17,533	NO_2_ Annual average estimates at residential address at birth	Wheezing (6 to 18 months)	No	NA
Rosa MJ et al., 2017 [12]	PROGRESS pregnancy cohort, Mexico	552	PM_2.5_ Daily exposure	Wheezing (birth up to 4 years)	No	No sensitive trimester
Aguilera I et al., 2013 [13]	Four birth cohorts of the INMA project, Spain	2199	NO_2_ Annual average estimates at residential address in pregnancy	Wheezing (birth up to 12–18 months)	No	NA

*PM*_*2.5*_ Particulate matter < 2.5 μm in diameter; *NO*_*2*_ Nitrogen dioxide*, NA* Not assessed

**Table 2:** Association between exposure to traffic related air pollutants in pregnancy and asthma development

References	Type of study, Country	Subjects, no	Pollutants and exposure assessment	Outcome	Positive association with the outcome	Sensitivity windows
Lavigne E et al., 2019 [14]	Registry-based birth cohort, Toronto, Canada	160,641	UFPs, PM_2.5_, NO_2_ Daily exposure	Asthma (birth up to < 6 years)	Yes	Second trimester
Jung CR et al., 2019 [15]	TMCHD registry-based birth cohort, Taiwan	184,604	PM_2.5_ Daily exposure	Asthma (birth up to 3–10 years)	Yes	Weeks 6–22
Lavigne E et al., 2018 [16]	Registry-based birth cohort, Ontario, Canada	222,864	PM_2.5_, NO_2_ Daily exposure	Asthma (birth up to < 6 years)	Yes	Second trimester
Pennington AF et al., 2018 [17]	KAPPA, registry-based birth cohort, Atlanta, USA	19,951	PM_2.5_, NO_x_, CO Annual average	Asthma (2 to 6 years)	Yes	NA
Lee A et al., 2018 [18]	ACCESS pregnancy cohort, Boston, USA	736	PM_2.5_ Daily exposure	Asthma (birth up to 6 years)	Yes	Weeks 19–23 in exposed to maternal prenatal stress
Bose S et al., 2017 [19]	ACCESS pregnancy cohort, Boston, USA	752	NO_3_^−^ Daily exposure	Asthma (birth up to 6 years)	Yes in boys exposed to prenatal maternal stress	7–19 and 33–40 weeks
Sbihi H et al., 2017 [20]	Registry-based birth cohort, Vancouver, Canada	65,254	NO_2_, PM_2.5_ Daily exposure aggregated over the pregnancy period	Asthma trajectories (birth up to 7–10 years)	Yes	NA
Sbihi H et al., 2016 [21]	Case-control nested in a registry-based birth cohort, Vancouver, Canada	Pre-schoolers: 6948 cases, 34,621 controls; School-age: 1711 and 8577	BC, CO, NO, NO_2_, PM_2.5_, PM_10_ Daily exposure aggregated over the pregnancy period	Asthma (birth up to 6–10 years)	Yes only in preschoolers and only for PM_10_	NA

*BC* Black carbon, *CO* Carbon monoxide, *PM*_*2.5*_ Particulate matter < 2.5 μm in diameter, *PM*_*10*_ Particulate matter < 10 μm in diameter; *NO*_*x*_ Nitrogen oxides, *NO* Nitric oxide, *NO*_*2*_ Nitrogen dioxide, *NO*_*3*_ Nitrate, *UFPs* Ultra-Fine Particles ≤ 0.1 μm in diameter; *NA* Not assessed

Wheezing development was evaluated in nine cohorts: four after exposure in utero [10–13] and five after exposure in the first 24 months of child’s life [13, 22–25] **(**Tables 3 and 4**).**

**Table 3:** Association between exposure to traffic related air pollutants in early life and wheezing development

References	Type of study, Country	Subjects, no	Pollutants and exposure assessment	Outcome	Positive association with the outcome
Rancière F et al., 2017 [22]	PARIS birth cohort, France	2015	NO_x_ Exposure assessed in the first year of life	Wheezing phenotypes (birth up to 4 years)	Yes only for persistent wheezing
Aguilera I et al., 2013 [13]	Four birth cohorts, INMA project, Spain	2199	NO_2_ Annual average exposure estimated at address in the first year of life	Wheezing (birth up to 12–18 months)	No
Gehring U et al., 2010 [23]	PIAMA birth cohort, the Netherlands	3863	PM_2.5_, NO_2_, Soot Annual average exposure estimated at birth address	Wheezing phenotypes (birth up to 8 years)	Yes only for PM_2.5_ and early transient and late onset wheezing
Nordling E et al., 2008 [24]	Birth cohort, Sweden	3515	PM_10_, NO_x_ Annual average exposure estimated at address in the first year of life	Wheezing phenotypes (birth up to 4 years)	Yes only for NO_x_ and persistent wheezing.
Morgenstern V et al., 2007 [25]	GINI/LISA birth cohorts, Munich, Germany	3577	PM_2.5_ mass, PM_2.5_ absorbance, NO_2_ Annual average exposure estimated at birth address	Wheezing (birth up to 2 years)	No
Brauer M et al., 2002 [26]	PIAMA birth cohort, the Netherlands	3730	PM_2.5_, NO_2_, Soot Annual average exposure estimated at birth address	Wheezing (birth up to 2 years)	No

*PM*_*2.5*_ *P*articulate matter < 2.5 μm in diameter*, PM*_*10*_ particulate matter < 10 μm in diameter*, NOx Nitrogen oxides, NO Nitric oxide, NO*_*2*_ *Nitrogen dioxide*

**Table 4:** Association between exposure to traffic related air pollutants in early life and asthma development

References	Type of study, Country	Subjects, no	Pollutant and exposure assessment	Outcome	Positive association with the outcome
To T et al., 2020 [27]	T-CHEQ registry-based birth cohort, Ontario, Canada	1286	PM_2.5_, NO_2_ Average exposure assessed in the first 3 years of life	Asthma (birth up to 15–20 years)	Yes only for NO_2_
Jung CR et al., 2019 [15]	TMCHD registry-based birth cohort, Taiwan	184,604	PM_2.5_ Exposure assessed in the first year of life	Asthma (birth up to 3–10 years)	Yes
Lavigne E et al. 2018 [16]	Registry-based birth cohort Ontario, Canada	222,864	PM_2.5_, NO_2_ Exposure assessed in the first year of life	Asthma (birth up to < 6 years)	Yes only for NO_2_
Pennington AF et al., 2018 [17]	KAPPA registry- based birth cohort, Atlanta, USA	23,100	PM_2.5_, NO_x_, CO Annual average exposure estimated in the first year of life	Asthma (2 to 6 years)	Yes
Rancière F et al., 2017 [22]	PARIS birth cohort, France	2015	NOx Exposure assessed in the first year of life	Asthma (birth up to 4 years)	Yes
Tétreault L-F et al., 2016 [28]	Registry-based birth cohort, Quebec, Canada	1,183,865	PM_2.5_, NO_2_ Annual average exposure estimated at birth address	Asthma (birth up to 1–12 years)	Yes
Gehring U et al., 2015 [30]	PIAMA birth cohort, the Netherlands	3702	PM_2.5_ abs, PM_2.5_, PM_10_, PM coarse, NO_2_, elemental composition of PM_2.5_ and PM_10_ Annual average exposure estimated at birth address	Asthma (birth up to 11 years)	Yes only for NO_2_, K PM_2.5_, K PM_10_, S PM_2.5_, Zn PM_10_
Gehring U et al., 2015 [29]	BAMBSE, GINI plus, LISA plus and PIAMA birth cohorts, Sweden, Germany, the Netherlands	14,126	PM_2.5_ abs, PM_2.5_, PM_10_, PM coarse, NO_2_ Annual average exposure estimated at birth address	Asthma (birth up to 14–16 years)	Yes only for NO_2_, PM_2.5_ abs
Ranzi A et al., 2014 [31]	GASPII birth cohort, Italy	672	NO_2_ Annual average exposure estimated at birth address	Asthma (birth up to 7 years)	No
Fuertes E et al., 2013 [32]	GINI plus and LISA plus birth cohorts, Germany	6604	PM_2.5_ mass, PM_2.5_ abs, NO_2_ Annual average exposure estimated at birth address	Asthma (birth up to 10 years)	No
Gruzieva O et al., 2013 [33]	BAMSE birth cohort, Sweden	3633	NO_x_, PM_10_ Annual average exposure estimated at birth address	Asthma (birth up to 12 years)	Yes
Lindgren A et al., 2013 [34]	Registry-based birth cohort, southern Sweden	7898	NO_x_ Annual average exposure estimated at birth address	Asthma (birth up to 1–6 years)	Negative association
Clark NA et al., 2010 [35]	Case-control study, nested in a cohort (administrative databases), British Columbia, Canada	3482 cases, 17,410 controls	PM_2.5_, PM_10_, NO, NO_2_, CO, BC Exposure estimated at address in the first year of life	Asthma (birth up to 36–59 months)	Yes only for NO_2_, BC, CO, PM_10_
Gehring U et al., 2010 [23]	PIAMA birth cohort, the Netherlands	3863	PM_2.5_, NO_2_, Soot Annual average exposure estimated at birth address	Asthma (birth up to 8 years)	Yes

*Abs* Absorbance*, BC* Black carbon*, CO* Carbon monoxide, *PM*_*2.5*_ Particulate matter < 2.5 μm in diameter, *PM*_*10*_ *Particulate matter < 10* μm *in diameter, NO*_*x*_ *Nitrogen oxides, NO* Nitric oxide*, NO*_*2*_ Nitrogen dioxide, *NO*_*3*_ Nitrate*, UFPs Ultra-Fine Particles ≤ 0.1 μm in diameter, NA* Not assessed

A large variability in the air pollutants studied and in the methods of exposure assessment was observed across studies. (supplementary Tables 2 and 3) The most common traffic-related air pollutant markers were PM (PM_10_, PM_2.5_, PM coarse, and PM_2.5_ abs) and NO_2._ A few studies considered also other pollutants: NOx, NO_3_^−^, CO and UFPs.

We observed a moderate variability in the methods for exposure assessment among studies that considered PM; most of the studies published in the last 5 years used models based on satellite data with a spatial resolution of 1-km^2^, considering a complex and flexible modelling approach (supplementary Tables 2 and 3). For less recent studies on PM and for most of the studies on NO_2_ the most common method for exposure assessment was Land use regression (LUR) model. One study assessed exposure to NO_3_^−^ in pregnancy using a hybrid model of a chemical transport model (GEOS-Chem) and land-use regression [19]. Two studies during pregnancy [17, 21] and eight in the first 2 years after delivery [17, 22–24, 26, 33–35] studied exposure to NOx, NO, CO, BC, soot and EC attributed to traffic (ECAT) applying different methods for exposure assessment (supplementary Tables 2 and 3).

The variability in terms of pollutants, exposure assessment methods, and exposure levels chosen to present the results (e.g. interquartile range increase, mean or median levels etc.) as reported in detail in supplementary Tables 2 and 3 did not allow to do a meta-analysis.

Data on study quality are presented in supplementary Tables 4 and 5.

Regarding the “Selection” items, all the studied cohorts were considered representative of the general population, as cohorts of susceptible populations were excluded.

In cohorts evaluating exposure in pregnancy the outcome of interest (wheezing or asthma in offspring) was, by definition, not present at the beginning of the study. Conversely, in all except one study [13, 15, 16, 22–35] which evaluated exposures in early life, there was an overlap between the period of exposure measurement and that of outcome development. This might represent a relevant risk of bias, especially for studies in which the outcome of interest was wheezing evaluated in the first few months/years of life.

Regarding the “Comparability” domain (supplementary Tables 4 and 5), except for five studies assessing in utero exposure [17–21] and two studies assessing exposure in early life [17, 28] all the other studies adjusted for both second-hand smoking and asthma predisposition, important potential confounders of the association between exposure to traffic-related air pollutants and wheezing and asthma. Five of 12 studies on pregnancy exposure to air pollutants adjusted for exposure during early life [10, 12, 14–16] while only three of 16 studies on early life exposures also accounted for it in their analysis exposure during pregnancy [15, 16, 35]. Moreover, several cohorts considered - often in sensitivity analyses - also changes of home address for a more precise evaluation of exposure to air pollutants [11, 13–21, 23, 26–32, 34, 35].

As for the “Outcome” domain (supplementary Tables 4 and 5), we defined that a follow-up of 2 and of 6 years was long enough to detect the occurrence of wheezing and asthma, respectively. According to this definition, for exposure in pregnancy follow-up was not long enough for wheezing or asthma to occur in two [11, 13] and two cohorts [15, 17], respectively. For exposures in the first 2 years of life follow up was not long enough for wheezing to occur in all the subjects in one cohort [13] and for asthma in four cohorts [15, 17, 34, 35].

Only three cohorts had a follow-up rate ≤ 60%, considered as likely to introduce a bias [17, 22, 31].

Tables 1 and 2 and supplementary Table 2 provide a summary of the 12 studies evaluating the association between exposure to traffic-related air pollutants in pregnancy and wheezing and asthma development [10–21].

The sample sizes ranged from 552 to 222,864, being the largest cohorts based on registries. Most of the studies evaluated exposures to particulate matter (9/12 studies) and eight to gases including NO_2_ (six studies), NOx, NO_3_^−^, NO, and CO.

Follow-up periods varied according to the outcome, ranging from 6 to 48 months for wheezing and from 2 to 10 years for asthma, though in the majority of studies on asthma incidence children were followed up at least up to school age.

Only 4 studies examined the development of wheezing after exposure to traffic-related air pollutants in pregnancy [10–13]. One study (GUSTO birth cohort, Singapore; 953 subjects) [10] reported an association between PM_2.5_ measured at eight stations and wheezing in the first 2 years of life. This was not confirmed in another small birth cohort (PROGRESS pregnancy cohort, Mexico; 552 subjects) [12]. No association was found for exposure to NO_2_ in pregnancy either in the INMA birth cohort in Spain (2199 subjects) [13] and in the MoBa pregnancy cohort in Norway [11]; this was a large cohort (17.533 subjects) exposed to low levels of NO_2_ (mean: 13.6 μg/m3).

Conversely, a positive association between exposure to both particulate and gases during pregnancy and asthma development was found in all the studies.

Five studies tried to identify “sensitive time periods” for exposure to air pollutants during the prenatal period and asthma development [14–16, 18, 19]. A sensitive window was found in four studies [14–16, 18] in the second trimester of pregnancy (weeks 13 to 24) for exposures either to UFP, PM_2.5_, or to NO_2_. Notably, the susceptibility during this sensitive window seemed to be more critical for boys with elevated maternal stress during gestation [18].

Tables 3 and 4 and supplementary Table 3 describe the 19 studies [13, 15–17, 23–26, 28–35] evaluating the association between exposure to traffic-related pollutants in the first 2 years of children’s life and wheezing and asthma development.

The sample sizes ranged from 672 to 1.183.865 subjects. Seventeen studies evaluated exposures to gases and 14 to PM.

Follow-up periods varied according to the outcome, being from 12 months to 8 years for wheezing and from 12 months to 16 years for asthma.

Three studies that followed children up to 4–8 years of life focused on wheezing phenotypes (Table 3): two found an association between exposure to NOx and persistent wheezing [22, 24] and one between PM_2.5_ and early transient and late-onset wheezing [23]. No association was found in three studies that evaluated exposure to NO_2_ or PM_2.5_ and wheezing in the first 2 years of children’s life with no mention of phenotypes [13, 25, 26].

Eleven [15–17, 22, 23, 27–30, 33, 35] of 14 studies found an association with exposure to one or more pollutants at the birth address or in the first year(s) of life and development of asthma. **(**Table 4**)** A positive association with asthma incidence was found more often for NO_2_ and PM_2.5_. One study performed in Italy [31] on a small cohort (672 subjects) did not find an association between exposure to NO_2_ measured at the birth address and development of asthma in the first 7 years of life. A study in the GINA plus and LISA plus birth cohorts (6604 subjects) [32] also did not find an association between exposure to PM_2.5_ and NO_2_ at the birth address and asthma incidence from birth up to 10 years. However, in another study [29] where data from the same cohorts collected over a longer follow-up period (14 to 16 years) were put together to those of other larger cohorts (BAMSE and PIAMA) and meta-analyzed, an association was found for NO_2_ and PM_2.5_. Finally, Lindgren and colleagues [34] found a negative association between exposure to NOx at birth and the development of asthma in children aged 2 to 6 years, though the study, also according to authors, might have been subjected to several biases.

## Discussion

Our systematic review summarized current published evidence from prospective unselected cohort studies on the association between exposure to traffic-related air pollutants in the first 1000 days of life -including pregnancy and the first 2 years after birth- and the subsequent risk of developing asthma and wheezing in childhood. We found consistent results for exposure to both NOx and PM in pregnancy and asthma development in childhood [14–21], with a more vulnerable window of exposure in the weeks corresponding to the second trimester of pregnancy [14–16, 18]. The susceptibility during this window of exposure seems to be modified by gender and stress-related factors; in fact, air pollution exposure during thesecond trimester of pregnancy (weeks 19–23) seems more critical in case of elevated maternal stress during gestation, particularly for male newborns [18].

The relationship between exposure to air pollutants in pregnancy and development of wheezing in childhood was evaluated in only four studies [10–13], and a significant association was found with exposure to PM_2.5_ in only one [10], while two studies did not find an association with exposure to NO_2_ [11, 13].

Also, for exposures to traffic-related air pollutants in the first 2 years after birth, the results were not concordant for wheezing development, while a positive association was found in most of the studies evaluating PM and NOx and the risk of asthma development [15–17, 23, 27–30, 33, 35].

As previously discussed, a large variability among studies in terms of pollutants considered, exposure assessment, and air pollutants levels, prevented us to perform a meta-analysis.

On the other hand, an accurate evaluation of the characteristics and the quality of the studies included in this systematic review gave interesting hints and allowed several important considerations.

The association found for exposure in pregnancy and asthma at school age is concordant with findings of an adverse impact of prenatal air pollution exposure on lung function [36–38]. In three studies [14, 16, 19] the second trimester of pregnancy was identified as a vulnerable period for asthma development both for exposure to PM and NO_2_. In studies evaluating lung function, the evidence of a more vulnerable trimester is weaker, though two studies also mentioned the second trimester [38, 39]. A recent Editorial [40] on inconclusive results on the most vulnerable time-period of exposure in pregnancy for lung function outcome in childhood pointed out methodological issues, highlighting the need of a more precise exposure assessment and statistical methods able to identify weeks of gestation rather than specific trimesters. In four studies included in our review [14–16, 18] which identified the second trimester of pregnancy as a vulnerable period, daily exposures were available, and distributed lag nonlinear models were used to identify susceptible weeks, thus allowing a precise definition of time windows of exposure. The availability of only two studies based on small birth cohorts [10, 12] evaluating the association between intrauterine PM_2.5_ exposure and wheezing in offspring as the outcome, and which found opposite results, does not permit to derive any conclusion. Exposure to LUR-modelled prenatal traffic-related NO_2_ was also evaluated in two larger birth cohorts [11, 13] and no association was found for the development of wheezing in the first 18 months of life. Mean NO_2_ exposures in the two cohorts were quite different being 39.1 μg/m^3^ for the INMA cohort and only 13.6 μg/m^3^ for the MoBa cohort, in this case largely below the EU air quality standard of 40 μg/m^3^. The fact that wheezing incidence in early childhood was not associated with in utero exposure to traffic related air pollutants, whereas asthma incidence at school age was, allows several considerations: the lack of large studies and hence a problem of potency, the fact that wheezing in childhood and asthma are different disease entities or latency in disease manifestation.

There is little doubt on the relationship between acute exposure to high levels of air pollution and increased respiratory symptoms in children, including cough and wheeze, and visits to emergency departments for respiratory illnesses [7]. Whether there is also an association between early postnatal exposure to air pollution and wheezing and asthma development is a more contentious issue. In our systematic review an association between exposure to gases, in particular to NO_2_, but also in a number of studies to PM, in particular to PM_2.5_, and asthma incidence has been reported in most of the studies.

In their systematic review and metanalysis, Bowatte and colleagues [8] concluded that exposure to traffic-related air pollutants (NO_2_, PM_2.5,_ and BC) from birth up to 5 years of age was associated with new onset of asthma throughout childhood. The association found between exposure to NO_2_ in the five studies meta-analysed was modest (OR 1.09; 95% CI 0.96 to 1.23 per 10 mcg/m^3^ increase) with a high heterogeneity between the studies. Association between PM_2.5_ (four studies) and BC (only three studies) and asthma incidence was slightly higher with an OR 1.14 (95% CI 1.00 to 1.30) per 2 μg/m^3^ increase and OR 1.20 (95% CI 1.05 to 1.38) per 1 × 10 ^− 5^ m^− 1^ increase, respectively. Only few studies in the review of Bowatte and colleagues are included also in the present study, the others being on selected cohorts or evaluating exposure to pollutants beyond the first 2 years of children’s life, raising a problem of overlap between the period of exposure measurement and that of outcome development. Among the more recent studies in our review (Tables 3 and 4 and supplementary Table 3), the association is expressed per one IQR increase of the air pollutants and a formal comparison among these studies and the older ones is difficult. Other methodological issues that could affect comparability among studies in our review are exposure models and age at outcome measurement. While more recent studies used models based on satellite data [12, 14–16, 18, 19, 28], allowing to obtain daily data and hence reliable exposure estimates in the first one or 2 years of life, studies published before 2015 mostly considered an average annual exposure estimated at the birth address. Furthermore, in most of these studies, exposure models based on air pollution measurements taken in different sampling campaigns done during several periods of one/two weeks and then averaged to represent annual mean were used to assess exposure to air pollution at the birth address, and this could represent a problem for the assessment of a narrow exposure period like the first one or 2 years of life.

As for children age at asthma diagnosis, a study by Gehring et al. [29] aimed at assessing the longitudinal associations between exposure to air pollution and development of asthma, noticed that the effects of air pollution on asthma incidence were larger after the age of 4 years, where asthma diagnosis is more likely to be made. Though most of the studies in our review evaluating the association between air pollution exposure in the first 2 years of life and asthma incidence followed children up to school age, in some [14–21, 23, 27–34] a follow-up and hence asthma diagnosis was limited to the first years of life in all or in part of the subjects studied. This resulted also in a partial overlapping between the period of exposure and the development of the outcome. As already discussed, this overlap is more critical for studies that evaluated wheezing as an outcome. Interestingly, in two [22, 24] of the three studies [22–24] that evaluated wheezing phenotypes, there was an association between exposure to NOx and persistent wheezing at 4 years of life, a condition often associated with asthma.

## Conclusions

Traffic-related air pollution during pregnancy increases the risk of asthma development among children and adolescents. This is in line with studies that considered lung function as an outcome. Also, in line with part of the studies on lung function is the finding of a susceptible time-window in the second trimester of pregnancy which corresponds to a period of intense airways development. We also confirmed a relationship between exposure in the first 2 years of life and asthma, although the time frame and hence the relationship between air pollutants exposure and asthma incidence needs to be further confirmed in studies with more precise exposure assessment. This is crucial for setting up more efficacious preventive strategies. Few studies with inconsistent results are available on the relationship between exposure to air pollutants either in pregnancy or in the 2 years after birth and wheezing development.

## Supplementary Information

**Additional file 1:** Supplementary Tables.

## Data Availability

All data are available within the article and supplemental material.

## Abbreviations

BC: Black Carbon
CI: Confidence Intervals
CO: Carbon Monoxide
EC: Elemental Carbon
ECAT: Elemental Carbon Attributed To Traffic
IQR: Interquartile Range
LUR: Land Use Regression
NO: Nitric Oxide
NO_2_: Nitrogen Dioxide
NOx: Nitric Oxides
O_3_: Ozone
PM: Particulate Matter
SO_2_: Sulphur Dioxide
UFPs: Ultra-Fine Particles
