# Supplementary material for: The first 1000 days of life: traffic-related air pollution and development of wheezing and asthma in childhood. A systematic review of birth cohort studies
Source: Environ Health. 2021 Apr 17;20:46. doi: 10.1186/s12940-021-00728-9 (PMC8053261; doi:10.1186/s12940-021-00728-9)
Supplement: Supplementary file 1 — Additional file 1: Supplementary Tables. [file 12940_2021_728_MOESM1_ESM.docx]

**Supplementary table 1.** Search strategy for Medline and Embase

|  | **Search strategy for Medline** | **Search strategy for Embase** |
| --- | --- | --- |
| Exposure | ((traffic[tiab] OR vehic*[tiab] OR environment*[tiab]) AND (pollut*[tiab] OR emission*[tiab] OR densit*[tiab])) OR (air[tiab] AND pollut*[tiab]) OR “Environmental Pollution”[Mesh] OR “sulphur dioxide”[tiab] OR “SO2”[tiab] OR “nitrogen oxide”[tiab] OR “nitrogen oxides”[tiab] OR “NOx”[tiab] OR “NO2”[tiab] OR “ozone”[tiab] OR “O3”[tiab] OR “nitrates”[Mesh] OR nitrate*[tiab] OR “diesel”[tiab] OR “particulate”[tiab] OR ((PM10[tiab] OR PM2.5) NOT (“prospective memory” [tiab] OR postmenopaus*[tiab] OR “phosphamide mustard” [tiab] OR “pregnancy morbidity”[tiab])) OR (“PAH”[tiab] NOT (“pulmonary arterial hypertension”[tiab] OR “Phenylalanine hydroxylase”[tiab])) OR “black carbon”[tiab] OR “elemental carbon”[tiab] OR “organic carbon”[tiab]) | ((traffic:ti,ab OR vehic*:ti,ab OR environment*:ti,ab) AND (pollut*:ti,ab OR emission*:ti,ab OR densit*:ti,ab)) OR (air:ti,ab AND pollut*:ti,ab) OR 'traffic pollution'/exp OR 'air pollution'/exp OR sulphur dioxide:ti,ab OR SO2:ti,ab OR nitrogen oxide:ti,ab OR nitrogen oxides:ti,ab OR NOx:ti,ab OR NO2:ti,ab OR ozone:ti,ab OR O3:ti,ab OR 'nitric acid derivative'/exp OR nitrate*:ti,ab OR diesel:ti,ab OR particulate:ti,ab OR ((PM10:ti,ab OR PM2.5) NOT (prospective memory:ti,ab OR postmenopaus*:ti,ab OR phosphamide mustard:ti,ab OR pregnancy morbidity:ti,ab)) OR (PAH:ti,ab NOT (pulmonary arterial hypertension:ti,ab OR Phenylalanine hydroxylase:ti,ab)) OR black carbon:ti,ab OR elemental carbon:ti,ab OR organic carbon:ti,ab |
| Outcome | “Respiratory function tests”[Mesh] OR “Respiratory Tract Diseases"[Mesh] OR “Respiratory Sounds” [Mesh] OR “respiratory sounds”[Mesh] OR wheez*[tiab] OR otitis*[tiab] OR asthma*[tiab] OR “respiratory infections”[tiab] OR (respiratory[tiab] AND infect*[tiab]) OR “lung function”[tiab] OR “lung development”[tiab] OR “lung health”[tiab] OR “bronchitis”[tiab] OR “broncopneumonia”[tiab] OR “laryngitis”[tiab] OR “rhinitis”[tiab] OR “cough”[tiab] OR “sinusitis”[tiab] OR “pneumonia”[tiab] | 'respiratory function'/exp OR 'respiratory function disorder'/exp OR wheez*:ti,ab OR otitis*:ti,ab OR asthma*:ti,ab OR respiratory infection:ti,ab OR (respiratory:ti,ab AND infect*:ti,ab) OR lung function:ti,ab OR lung development:ti,ab OR lung health:ti,ab OR bronchitis:ti,ab OR broncopneumonia:ti,ab OR laryngitis:ti,ab OR rhinitis:ti,ab OR cough:ti,ab OR sinusitis:ti,ab OR pneumonia:ti,ab |
| Population | "Pregnancy"[Mesh] OR pregnan*[tiab] OR gestation*[tiab] OR "Fetus"[Mesh] OR “foetus”[tiab] OR “fetus”[tiab] OR “fetal”[tiab] OR “foetal”[tiab] OR intrauterine*[tiab] OR uterin*[tiab] OR perinatal*[tiab] OR “prenatal”[tiab] OR "Infant, Newborn"[Mesh] OR neonat*[tiab] OR newborn*[tiab] OR “toddler”[tiab] OR pediatr*[tiab] OR paediatr*[tiab] OR preschool*[tiab] OR child*[tiab] OR "Child"[Mesh] OR "Infant"[Mesh] OR Infant*[tiab] OR teen*[tiab] OR "Adolescent"[Mesh] OR adolescen*[tiab] | ([embryo]/lim OR [fetus]/lim OR [newborn]/lim OR [infant]/lim OR [child]/lim OR [preschool]/lim OR [school]/lim OR [adolescent]/lim OR pregnancy OR pregnant OR perinatal OR gestation*) |

**Supplementary table 2**. Association between exposure to traffic related air pollutants in pregnancy and wheezing and asthma development

| **Reference** | **Type of study, Country,**  **Sample size** | **Pollutant, Exposure levels** | **Exposure assessment** | **Outcome** | **Results** |
| --- | --- | --- | --- | --- | --- |
| Lavigne E et al, Am J Respir Crit Care Med, 2019  [14] | Registry-based birth cohort, Toronto, Canada;  N=160,641 | **Pollutants**: UFPs, PM_2.5_, NO_2_  **IQR levels**: 10,820 count/cm3 for UFPs, 3.8 µg/m^3^ for PM_2.5_, 9.7 ppb for NO_2_ | PM_2.5_: satellite data available at a 1*1km resolution. UFPs and NO_2_: LUR model | Asthma: primary care visits/ hospitalizations.  From birth up to <6 years | UFPs, PM_2.5_ and NO_2_ concentrations during the second trimester were positively associated with childhood asthma incidence; HR:1.09; 95% CI 1.06–1.12 (for a 10,770 count/cm^3^ [IQR] increase), HR:1.08; 95% CI 1.05–1.11 (for a 3.8 mg/m^3^ [IQR] increase) and HR:1.12; 95% CI 1.09–1.15 (for a 9.7 ppb [IQR] increase) respectively |
| Jung CR et al, J Allergy Clin Immunol, 2019 [15] | TMCHD registry-based birth cohort, Taiwan;  N=184,604 | **Pollutant:** PM_2.5_  **Mean level**: 36.2 +/-3.5 µg/m^3^ | PM_2.5_: 10-km resolution hybrid model based on satellite data | Asthma: primary care visits/ hospitalizations.  From birth up to 3-10 years | PM_2.5_ (10 µg/m^3^ increase): HR 1.26 (1.17-1.35).  Stronger association during gestational weeks 6 to 22.  No association after adjustment for exposure in the first year of life |
| Lavigne E et al, Eur Respir J, 2018  [16] | Registry-based birth cohort, Ontario, Canada;  N=222,864 | **Pollutants:** PM_2.5_, NO_2_  **IQR levels**: in the second trimester: 3.7 µg/m^3^, 8.6 ppb | PM_2.5_: satellite data available at 1*1 km resolution; NO_2_: LUR model | Asthma: primary care visits/ hospitalizations  From birth up to < 6 years | PM_2.5_, NO_2_ (IQR increase in the second trimester): HR 1.07 (1.06-1.09), HR 1.06 (1.03-1.08) |
| Pennington AF et al, Epidemiology, 2018  [17] | KAPPA registry-based birth cohort, USA;  N=23,100 | **Pollutants:**PM_2.5_, NO_x_, CO  **Median levels**: 1.49 µg/m^3^, 61.4 ppb, 0.62 ppm respectively | PM_2.5_, NO_x_, CO: Research LINE-source dispersion model for near-surface releases (RLINE) at 250 m resolution | Asthma: asthma diagnosis and dispensing medication.  From 2 up to 6 years | PM_2.5_, NOx, CO (risk difference per 2.7-fold natural log increase): RD 3.5% (0.6-6.5), RD 3.1% (0.3-5.9), RD 3.1% (0.0-6.1) respectively . |
| Soh S et al, Int J Environ Res Public Health, 2018  [10] | GUSTO birth cohort, Singapore;  N=953 | **Pollutant:**PM_2.5_  **Quartiles**: Q2 16.84 µg/m^3^, Q3 17.59 µg/m^3^, Q4 19.07 µg/m^3^ | PM_2.5_: average measured from eight stations with representation from urban stations | Wheezing: parental reporting.  From birth up to 2 years | PM_2.5_ (for Q2, Q3 and Q4): IRR 1.50 (1.11-2.03), IRR 1.56 (1.17-2.08), IRR 1.38 (0.96-1.99)  No consistent associations per trimesters of pregnancy |
| Lee A et al, J Allergy Clin Immunol, 2018 [18] | ACCESS pregnancy cohort, USA;  N=736 | **Pollutant:**PM_2.5_  **Median level (IQR):** 11.2 (10.2-11.9) µg/m^3^ | PM_2.5_: spatiotemporal model based on satellite data with a 10*10 km spatial resolution | Asthma; maternal reporting.  From birth up to 6 years | PM_2.5_ (IQR increase): OR 1.17 (1.04-1.30) Sensitive windows at 19-23 weeks in exposed to prenatal maternal stress, OR 1.15 (1.03-1.26) |
| Madsen C et al, BMJ Open, 2017  [11] | MoBa pregnancy cohort, Norway;  N=17,533 | **Pollutant:**NO_2_  **Mean level** **(SD):** 13.6±6.9 µg/m^3^ | NO_2_: LUR model | Wheezing: maternal reporting.  From 6 to 18 months | No association. |
| Bose S et al, Am J Resp Crit Care Med, 2017 [19] | ACCESS pregnancy cohort, USA;  N=752 | **Pollutant:**NO_3_^-^  **Median level (IQR):** 1.30 (1.11-1.47) µg/m^3^ | NO_3_^-^: hybrid model of a chemical transport model (GEOS-Chem) and land-use regression term to make predictions at 1*1km grid cells | Asthma: maternal reporting.  From birth up to 6 years | No association overall.  Ln NO_3_^-^ (IQR increase): OR 1.95 (1.11-3.46) only in boys exposed to prenatal maternal stress. |
| Sbihi H et al, Am J Respir Crit Care Med, 2017  [20] | Registry-based birth cohort, Vancouver, Canada;  N=65,254 | **Pollutants:**NO_2_, PM_2.5_  **Median levels:** 33.3 µg/m^3^, 12.5 µg/m^3^ respectively | NO_2_ and PM_2.5_: LUR models with monitoring stations used to compute inverse-distance weighted averages of the three closest monitors within 50 km providing daily estimates | Asthma trajectories (late-onset chronic, early-onset chronic, transient):primary care visits/ hospitalizations.  From birth up to 7-10 years | NO_2_ (IQR increase) and early and late-onset chronic asthma: RRR: 1.51 (1.21-1.88), RRR 1.20 (1.03-1.41).  PM_2.5_ (IQR increase) and transient and late onset asthma: RRR 1.24 (1.08-1.42), RRR1.24 (1.07-1.45) |
| Rosa MJ et al, Allergy Asthma Immunol, 2017 [12] | PROGRESS pregnancy cohort, Mexico;  N=552 | **Pollutant:**PM_2.5_  **Median level (IQR):** 22.0 µg/m^3^ (18.9-25.7), 21.1 µg/m3 (18.8-25.6), 22.5 µg/m^3^ (19.0-27.3) for each trimester respectively | PM_2.5_: hybrid satellite-based method at a 1_*_1km spatial resolution | Wheezing: maternal reporting.  From birth up to 4 years | No association |
| Sbihi H et al, Eur Respir J, 2016  [21] | Case-control nested in a registry-based birth cohort, Vancouver, Canada;  Pre-schoolers: N=6,948 cases, N=34,621 controls;  School-age: N=1,711 and N=8,577 | **Pollutants:**PM_2.5_, NO_2_, NO, BC, PM_10_, CO  **IQR levels for cases**:  1.4 µg/m^3^;  9.4 µg/m^3^;  14.5 µg/m^3^;  1.2 10^-5^/m;  1.3 µg/m^3^;  165.0 µg/m^3^ respectively | PM_2.5_, NO_2_, NO, BC: LUR models**§**;  PM_10_, CO: monitoring stations to compute the inverse-distance weighted (IDW) averages from the three closest monitors within 50 km | Asthma: primary care visits/ hospitalizations  From birth up to 5 years (pre-schoolers) and from 6 up to 10 years (school age) | Pre-schoolers:  PM10 (IQR increase): OR 1.12 (95% CI 1.05;1.19)  No association for BC, CO, NO, NO_2_ and PM2.5  School age children:  Negative association for CO: OR 0.90 (0.83-0.98)  No association for other pollutants |
| Aguilera I et al, Environ Health Persperct, 2013 [13] | Four birth cohorts of the INMA project (Asturias, Gipuzkoa, Sabadell, Valencia), Spain;  N=2,199 | **Pollutant:** NO_2_  **IQR level:** 16.5 µg/m^3^ | NO_2_: LUR model | Wheezing: parental reporting.  From birth up to 12-18 months | No association |

*BC=black carbon; CI=confidence interval; CO=carbon monoxide; HR=hazard ratio; IQR=interquartile range; IRR= incidence rate ratio; LUR=land use regression model; PM_2.5_=particulate matter <2.5 μm in diameter; PM_10_= particulate matter <10 μm in diameter; NO_x_= nitrogen oxides;* *NO=nitric oxide; NO_2_=nitrogen dioxide; NO_3_^-^=nitrate;* *OR=odds ratio; Q=quartile; RD=risk difference; UFPs=ultra-fine particles ≤ 0.1 μm in diameter*

***§*** *Air pollutants exposure was assessed by both LUR and inverse-distance weighted models (IDW); only associations estimated by LUR models were considered, as this methods is more accurate*.

**Supplementary table 3.** Association between exposure to traffic related air pollutants in early life and wheezing and asthma development

| **Reference** | **Type of study, Country,**  **Sample size** | **Pollutant, Exposure levels** | **Exposure assessment** | **Outcome** | **Results** |
| --- | --- | --- | --- | --- | --- |
| To T et al, Eur Respir J, 2020  [27] | T-CHEQ registry-based birth cohort,  Ontario, Canada;  N=1,286 | **Pollutants**: NO_2_, PM_2.5_  **Median level (IQR):**  26.53 (24.38-28.52) ppb, 11.00 (10.13-11.80) mg/m^-3^ respectively | NO_2_: LUR PM_2.5_:  total column aerosol depth retrievals from MODIS using the GEOS-Chem chemical transport model | Asthma: ≥1 hospital admission or ≥2 asthma insurance claims for physician services in 2 consecutive years (the first claim is diagnosis date).  From birth up to 15-20 years | NO_2_ (for IQR increase): HR 1.17 (1.05–1.31).  No association for PM_2.5_. |
| Jung CR et al, J Allergy Clin Immunol, 2019  [15] | TMCHD registry-based birth cohort, Taiwan; N=184,604 | **Pollutant**: PM_2.5_  **Mean level (SD):**  35.6 +/- 3.5µg/m^3^ | PM_2.5_: 10-km resolution hybrid model based on satellite data | Asthma: primary care visits/hospitalizations  From birth up to 3-10 years | PM_2.5_ (for increase of 10 µg/m3 in the first year of life): HR 1.61 (1.48-1.76). |
| Lavigne E et al, Eur Respir J, 2018  [16] | Registry-based birth cohort, Ontario, Canada;  N=222,864 | **Pollutants**: PM_2.5_, NO_2_  **IQR levels:** 3.6 µg/m^3^, 8.9 ppb | PM_2.5_: satellite data available at 1*1km resolution;  NO_2_: LUR model | Asthma: primary care visits/ hospitalizations.  From birth up to < 6 years | NO_2_ (for IQR increase):  HR 1.08 (1.06–1.09).  No association for PM_2.5_. |
| Pennington AF, Epidemiology, 2018  [17] | KAPPA registry-based birth cohort, Atlanta, USA; N=23,100) | **Pollutants**: PM_2.5_, NOx, CO  **Median levels:** 1.41 µg/m^3^, 55.5 ppb, 0.59 ppm respectively | PM_2.5_, NOx, CO:  Research LINE-source dispersion  model for near-surface releases (RLINE) at 250m resolution | Asthma: asthma diagnosis and dispensing medication.  From 2 up to 6 years | PM_2.5_, NOx, CO (natural log increase): RD 3.5% (0.5-6.4), RD 3.1% (0.3-5.9), RD 3.1% (0.1-6.2). |
| Rancière F et al, Environ Health Perspect, 2017 [22] | PARIS birth cohort, France;  N=2,015 | **Pollutant**: NOx  **Median level (IQR):**  75 (66-89) µg/m^3^ | NOx: extra index based on an air dispersion model adapted from the Danish Operational Street Pollution Model (OSPM) | Wheezing phenotypes (early-transient, late-onset, persistent) and asthma: parental questionnaire  Form birth up to 4 years | NOx (for IQR increase of 26 μg/m^3^ NO_2_ equivalent) and persistent wheezing or asthma: OR 1.27 (95% CI 1.09-1.47), OR 1.15 (1.01-1.31) respectively.  No association with early-transient and late-onset wheezing |
| Tétreault LF et al, Environ Health Perspect, 2016 [28] | Registry-based birth cohort, Quebec, Canada;  N=1,183,865 | **Pollutants**:NO_2_, PM_2.5_  **IQR levels:** 5.45ppb, 6.50 µg/m^3^ respectively | NO_2_: LUR model estimated on a 5x5 m grid for the island of Montreal PM_2.5_: satellite imagery converted into surface levels using the global chemical transport model GEOS-Chem at 10x10km grid | Asthma: physician visits/hospitalizations  Form birth up to 1-12 years | NO_2_, (IQR increase at the birth address): HR 1.04 (1.02-1.05),  PM_2.5_ (IQR increase at the birth address) HR 1.31 (1.28-1.33). |
| Gehring U et al, Epidemiology, 2015  [30] | PIAMA birth cohort, the Netherlands;  N=3,702 | **Pollutants**:PM_2.5_ abs, PM_2.5_, PM_10_, PM _coarse_, NO_2_ and concentrations of copper, iron, potassium, nickel, sulfur, silicon, vanadium, and zinc in PM_2.5_ and PM_10_  **IQR levels:** 0.29*10^-5^m^-1^ (PM_2.5_ abs); 1.2μg/m^3^ (PM_2.5_); 1.2 μg/m^3^ (PM_10_); 0.8 μg/m^3^ (PM _coarse_); 8.4 μg/m^3^ (NO_2_); 1.5 ng/m^3^ (Cu PM_2.5_); 4.8 ng/m^3^ (Cu PM_10_); 34.8 ng/m^3^ (Fe PM_2.5_); 142.1 ng/m^3^ (Fe PM_10_); 6.9 ng/m^3^ (K PM_2.5_); 15.8 ng/m^3^ (K PM_10_); 1.2 ng/ m^3^ (Ni PM_2.5_); 1.3 ng/m^3^ (Ni PM_10_); 114.5 ng/m^3^ (S PM_2.5_); 74.9 ng/m^3^ (S PM_10_); 23.9 ng/m^3^ (Si PM_2.5_); 92.0 ng/m^3^ (Si PM_10_); 1.7 ng/m^3^ (V PM_2.5_); 2.0 ng/m^3^ (V PM_10_); 8.6 ng/m^3^ (Zn PM_2.5_); 13.4 ng/m^3^ (Zn PM_10_) | LUR models for all pollutants | Asthma: parental reporting of doctor diagnosis.  From birth up to 11 years | Associations (IQR increase) with incident asthma and exposures at birth address: NO_2_ - OR 1.13 (1.01-1.25); K PM_2.5_ - OR 1.13 (1.03-1.25); K PM_10_ - OR 1.13 (1.05-1.21); S PM_2.5_- OR 1.21 (1.01-1.44); Zn PM_10_ - OR 1.13 (1.02-1.25)  No association for other pollutants considered |
| Gehring U et al, Lancet Respir Med, 2015  [29] | BAMBSE, GINI and LISA plus, and PIAMA birth cohorts,  Sweden, Germany, the Netherlands;  N=14,126 | **Pollutants**: PM_2.5_ abs, PM_2.5_, PM_10_, PM coarse, NO_2_  **Median values (range):** 0.6-1.7 x10-5/m, 8.1-17.2 µg/m^3^, 15.5-25.2 µg/m^3^, 6.5-8.4 µg/m^3^, 12.5-23.2 µg/m^3^ respectively | LUR models for all pollutants  For NO_2_: three measurements (lasting two-weeks each) at 80/40 sites within 1 year; simultaneous measure of PM_2.5_, PM_10_, PM _coarse_ and soot in half sites | Asthma: parental reporting of at least 2: (1) doctor diagnosed asthma ever; (2) wheezing last 12 months; (3) asthma medication last 12 months.  From birth up to 14-16 years | NO_2_ (10 μg/m^3^ increase): OR 1.13 (1.02-1.25).  PM_2.5_ abs (1unit increase): 1.29 (1.00 –1.66)  No association for PM_2.5_, PM_10_, and PM coarse |
| Ranzi A et al, Occup Environ Med, 2014  [31] | GASPII birth cohort, Italy;  N=672 | **Pollutant**:NO_2_  **Mean level (SD):** 37.2 (7.2) µg/m^3^ | NO_2_: LUR model | Asthma: parental reporting of doctor diagnosis.  From birth up to 7 years | No association |
| Aguilera I et al, Environ Health Persperct, 2013  [13] | Four birth cohorts of the INMA project,  Spain (Asturias, Gipuzkoa, Sabadell, Valencia);  N=2,199 | **Pollutant**:NO_2_  **IQR level:** 15.7 µg/m^3^ | NO_2_: LUR model; ambient levels measured with passive samplers | Wheezing: parental reporting.  From birth up to 12-18 months | No association |
| Fuertes E. et al, Peer J, 2013  [32] | GINIplus and LISAplus, Germany;  N=6,604 | **Pollutants**:PM_2.5_ mass, PM _2.5_ abs, NO_2_  **IQR levels:** 4.0 µg/m^3^, 0.5 x10^-5^/m, 6.1 µg/m^3^ respectively | PM_2.5_ mass, PM _2.5_ abs, NO_2:_ LUR models | Asthma: parental reporting of doctor diagnosis.  From birth up to 10 years | No association |
| Gruzieva O et al, Epidemiology, 2013  [33] | BAMSE birth cohort,  Sweden;  N=3,633 | **Pollutants:** NO_x_, PM_10_  Percentile Difference (5^th^ to 95^th^): 7.2 and 46.8 µg/m^3^ respectively | NO_x_, PM_10_: emission inventories and dispersion models of the Stockholm and Uppsala Air Quality Management Association | Asthma: parental reporting.  From birth up to 12 years | PM_10_ (5^th^ to 95^th^ difference during the first year of life): OR 2.39 (1.18-4.86); NOx (5^th^ to 95^th^ difference during the first year of life): OR 1.87 (1.01-3.44) |
| Lindgren A et al, Environmental Health, 2013  [34] | Registry-based birth cohort, southern, Sweden;  N=7,898 | **Pollutant:** NO_x_  **Median level (IQR**): 17.6 (11.8-21.1) µg/m^3^ | NO_x_: Annual means were obtained from an emission database (EDB) for NOx with spatial resolution of 100×100 m | Asthma:(1) first ever dispensed asthma medication; (2) third year with dispensed asthma medication; (3) doctor diagnosed asthma.  From birth up to 1-6 years | Negative association with incident asthma: NO_x_ 15-25 μg/m^3^ OR 0.8 (0,7-0.9); NO_x_ >25 μg/m^3^ OR 0.7 (0,5-0.9). Similar associations also for use of asthma medications |
| Clark NA et al, Environ Health Perspect, 2010  [35] | Case-control study, nested in a registry-based birth cohort, British Columbia, Canada;  N=3,482 cases, N=17,410 controls | **Pollutants:** NO, NO_2_, CO, PM_10_, PM_2.5_, BC  **Mean level (SD)** **for cases**: 30.8 (12.7) µg/m^3^, 29.8 (5.5) µg/m^3^, 617.5 (132.5) µg/m^3^, 12.4 (1.0) µg/m^3^, 4.6 (2.4) µg/m^3^, 0.7 (0.3)10^-5^/m respectively | NO, NO_2_, PM_2.5_, BC: LUR models with a spatial resolution of 10*10m**§**;  CO, PM_10_: daily values at the three closest monitors within 50 km were weighted by their inverse distance to the postal code of interest | Asthma: primary care visits/hospitalizations  From birth up to 36-59 months | NO_2_ (10 µg/m^3^ increase) OR 1.13 (1.04-1.23); BC (10^-5^/m increase in filter absorbance) OR 1.14 (1.01-1.29)  CO (100 µg/m^3^ increase) OR 1.10 (1.06-1.13); PM_10_ (1 µg/m^3^ increase): OR 1.07 (1.03-1.12)  No association for other pollutants |
| Gehring U et al, Am J Respir Crit Care Med, 2010  [23] | PIAMA birth cohort,  the Netherlands; N=3,863 | **Pollutants:** PM_2.5_, NO_2_, Soot  **IQR levels**: 3.2 µg/m^3^, 10.4 µg/m^3^, 0.57 x10^-5^/m | PM_2.5_, NO_2_, Soot: LUR models.  Four measurements (lasting two-weeks each) at 40 sites within 1 year and then adjusted by temporal trends to calculate the long-term average concentrations | Wheezing phenotypes (early transient, late-onset, persistent) and asthma: parental reporting.  From 1 up to 8 years | PM_2.5_ (IQR increase) and early transient or late onset wheezing: OR 1.29 (1.04-1.62), OR 1.18 (1.01-1.37)  NO_2_ (IQR increase) and asthma: OR 1.19 (1.05 -1.34); PM_2.5_ (IQR increase) and asthma: OR 1.28 (1.10-1.49); Soot (IQR increase) and asthma: OR 1.21 (1.06 -1.38)  No association for the other exposures and outcomes studied |
| Nordling E et al, Epidemiology, 2008 [24] | BAMSE birth cohort, Sweden;  N=3,515 | **Pollutants:** PM_10_, NOx  **Percentile difference** ( 5^th^ to 95^th^): 6 µg/m^3^, 44 µg/m^3^ respectively. | PM_10_, NO_x_: emission inventory together with dispersion models to map outdoor levels of selected pollutants from selected emission sources over time at the relevant geographical locations | Wheezing overall and phenotypes (transient, persistent, late-onset): parental reporting.  From birth up to 4 years | NO_x_ (difference between the 5^th^ and 95^th^ percentile range) and persistent wheezing: OR 1.60 (1.09-2.36)  No association for the other exposures and outcomes studied |
| Morgenstern V et al, Occup Environ Med, 2007 [25] | GINI/LISA, Munich Germany;  N=3,577 | **Pollutants**: PM_2.5_ mass, PM_2.5_ abs, NO_2_  **IQR levels**: 1.04 µg/m^3^, 0.22 x10^-5^/m 5.7 µg/m^3^ respectively | PM_2.5_ mass, PM_2.5_ abs, NO_2_: regression-based exposure modelling based on measured data and exogenous information | Wheezing: parental reporting.  From birth up to 2 years | No association |
| Brauer M et al, Am J Respir Crit Care Med, 2002 [26] | PIAMA birth cohort,  the Netherlands;  N=3,730 | **Pollutants**:PM_2.5_, NO_2_, Soot  **IQR levels**: 3.2 µg/m^3^, 10.3 µg/m^3^, 0.54 x10^-5^/m respectively | PM_2.5_, NO_2_, Soot: modelling approach combining air pollution measurements with GIS information | Wheezing: parental reporting.  From birth up to 2 years | No association |

*Abs=absorbance; BC=black carbon; CI=confidence interval; CO=carbon monoxide; HR=hazard ratio; IQR=interquartile range; LUR=land use regression model; PM_2.5_=particulate matter <2.5 μm in diameter; PM_10_= particulate matter <10 μm in diameter; NOx= nitrogen oxides;* *NO=nitric oxide; NO_2_=nitrogen dioxide; NO_3_^-^=nitrate;* *OR=odds ratio; Q=quartile; RD=risk difference; UFPs=ultra-fine particles ≤ 0.1 μm in diameter*

**§** Air pollutants exposure was assessed by both LUR and inverse-distance weighted models (IDW); only associations estimated by LUR models were considered, as this methods is more accurate.

**Supplemental table 4.** Study quality according to the Newcastle-Ottawa Quality Assessment Scale; studies evaluating exposure in pregnancy.

|  | | Lavigne E et al, 2019 [14] | Jung CR et al, 2019 [15] | Lavigne E et al, 2018 [16] | Pennington AF et al, 2018 [17] | Soh S et al, 2018 [10] | Lee A et al, 2017 [18] Bose S et al, 2017 [19] | Madsen C et al, 2017 [11] | Sbihi H et al, 2017 [20] Sbihi H et al, 2016 [21] | Rosa MJ et al, 2017 [12] | Aguilera I et al, 2013 [13] |
| --- | --- | --- | --- | --- | --- | --- | --- | --- | --- | --- | --- |
| SELECTION | 1) Representativeness of the exposed cohort | | | | | | | | | | |
|  | a) truly representative of the average in the community | Yes* | Yes* | Yes* | Yes* | Yes* | Yes* | Yes* | Yes* | Yes* | Yes* |
|  | b) partially representative;  c) selected groups;  d) no description |  |  |  |  |  |  |  |  |  |  |
|  | 2) Selection of the non-exposed cohort | | | | | | | | | | |
|  | a) drawn from the same community as the exposed cohort | Yes* | Yes* | Yes* | Yes* | Yes* | Yes* | Yes* | Yes* | Yes* | Yes* |
|  | b) drawn from a different source;  c) no description |  |  |  |  |  |  |  |  |  |  |
|  | 3) Ascertainment of exposure | | | | | | | | | | |
|  | a) secure record # | Yes * | Yes * | Yes * | Yes * | Yes * | Yes * | Yes * | Yes * | Yes * | Yes * |
|  | b) structured interview;  c) written self-report;  d) no description |  |  |  |  |  |  |  |  |  |  |
|  | 4) Demonstration that outcome of interest was not present at start of study | | | | | | | | | | |
|  | a) yes | Yes* | Yes* | Yes* | Yes* | Yes* | Yes* | Yes* | Yes* | Yes* | Yes* |
|  | b) no |  |  |  |  |  |  |  |  |  |  |
| COMPARABILITY | 1) Comparability of cohorts on the basis of the design or analysis | | | | | | | | | | |
|  | a) study controls for maternal smoking and history of asthma | Yes* | Yes* | Yes* | No (maternal asthma only) | Yes* | No (maternal smoking only) | Yes* | No (maternal smoking only) | Yes* | Yes* |
|  | b) study controls for early life exposure and/or changes of residence | Yes* | Yes* | Yes* | Yes* (changes of residence only) | Yes* (early life exposure only) | Yes* (changes of residence only) | Yes* (changes of residence only) | Yes * (changes of residence only) | Yes * (early life exposure only) | Yes* (changes of residence only) |
| OUTCOME | 1) Assessment of outcome | | | | | | | | | | |
|  | a) independent blind assessment |  |  |  |  |  |  |  |  |  |  |
|  | b) record linkage | Yes* | Yes* | Yes* | Yes* |  |  | Yes* | Yes* |  |  |
|  | c) self report |  |  |  |  | Parental reporting | Parental reporting |  |  | Parental reporting | Parental reporting |
|  | d) no description |  |  |  |  |  |  |  |  |  |  |
|  | 2) Was follow-up long enough for outcomes to occur | | | | | | | | | | |
|  | a) yes  (asthma diagnosis ≥ 6 years; wheezing ≥ 2 years) | Yes* |  | Yes* |  | Yes* | Yes* |  | Yes* | Yes* |  |
|  | b) no |  | No |  | No |  |  | No |  |  | No |
|  | 3) Adequacy of follow up of cohorts | | | | | | | | | | |
|  | a) complete follow up - all subjects accounted for at the last follow up | Yes* | Yes*  99.67% | Yes* |  |  |  |  |  |  |  |
|  | b) >60% subjects |  |  |  |  | Yes*  80% | Yes* 77.1% | Yes* 69.8% | Yes*  73% | Yes*  67.7% | Yes*  83.2% |
|  | c) ≤ 60% subjects accounted for |  |  |  | Yes  29.1% |  |  |  |  |  |  |
|  | d) no statement |  |  |  |  |  |  |  |  |  |  |

**Notes:** # see supplementary table 2 for ascertainment of exposure

**Supplementary table 5.** Study quality according to the Newcastle-Ottawa Quality Assessment Scale; studies evaluating early life exposure.

|  | | To T et al, 2020 [27] | | Jung CR et al, 2019 [15] | | Lavigne E et al, 2018 [16] | | Pennington AF et al, 2018 [17] | | Rancière F et al, 2017 [22] | | Tétreault LF et al, 2016 [28] | | Gehring U et al,2015 [30] Gehring U et al, 2010 [23] Brauer M et al, 2002 [26] |
| --- | --- | --- | --- | --- | --- | --- | --- | --- | --- | --- | --- | --- | --- | --- |
| SELECTION | 1) Representativeness of the exposed cohort | | | | | | | | | | | | | |
|  | a) truly representative of the average in the community | Yes* | | Yes* | | Yes* | | Yes* | | Yes* | | Yes* | | Yes* |
|  | b) partially representative;  c) selected groups;  d) no description |  | |  | |  | |  | |  | |  | |  |
|  | 2) Selection of the not exposed cohort | | | | | | | | | | | | | |
|  | a) drawn from the same community as the exposed cohort | Yes* | | Yes* | | Yes* | | Yes* | | Yes* | | Yes* | | Yes* |
|  | b) drawn from a different source;  c) no description |  | |  | |  | |  | |  | |  | |  |
|  | 3) Ascertainment of exposure | | | | | | | | | | | | | |
|  | a) secure record  # | Yes* | | Yes* | | Yes* | | Yes* | | Yes* | | Yes* | | Yes* |
|  | b) structured interview;  c) written self report;  d) no description |  | |  | |  | |  | |  | |  | |  |
| SELECTION | 4) Demonstration that outcome of interest was not present at start of study | | | | | | | | | | | | | |
|  | a) yes |  |  | |  | | Exposure in the first year and outcome (asthma) from 2 up to 6 years* | |  | |  | |  | |
|  | b) no | Exposure in the first 3 years of life; outcome (asthma) from birth up to 15-20 years. | Exposure in the first year and outcome (asthma) from birth up to 3-10 years | | Exposure in the first year and outcome (asthma) from birth up to < 6 years | |  | | Exposure in the first year and outcome (wheezing phenotypes and asthma) from birth up to 4 years | | Annual average exposure estimated at birth address and outcome (asthma) from birth up to 1-12 years | | Annual average exposure estimated at birth address and outcome from birth up to 11 years (asthma) and from birth up to 8 years (wheezing) | |
| COMPARABILITY | 1) Comparability of cohorts on the basis of the design or analysis | | | | | | | | | | | | | |
|  | a) study controls for maternal smoking and history of asthma | Yes* | Yes* | | Yes* | | No  (maternal asthma only) | | Yes* | | No | | Yes* | |
|  | b) study controls for early life exposure and/or changes of residence | Yes*  (change of residence only) | Yes* | | Yes* | | Yes*  (changes of residence only) | | No | | Yes*  (changes of residence only) | | Yes*  (changes of residence only) | |

| OUTCOME | 1) Assessment of outcome | | | | | | | |
| --- | --- | --- | --- | --- | --- | --- | --- | --- |
|  | a) independent blind assessment |  |  |  |  |  |  |  |
|  | b) record linkage | Yes* | Yes* | Yes* | Yes* |  | Yes* |  |
|  | c) self report |  |  |  |  | Parental reporting |  | Parental reporting |
|  | d) no description |  |  |  |  |  |  |  |
|  | 2) Was follow-up long enough for outcomes to occur | | | | | | | |
|  | a) yes  (asthma diagnosis ≥ 6 years; wheezing ≥ 2 years) | Yes* |  | Yes* |  | Yes* | Yes* | Yes* |
|  | b) no |  | No |  | No |  |  |  |
|  | 3) Adequacy of follow up of cohorts | | | | | | | |
|  | a) complete follow up - all subjects accounted for at the last follow up | Yes* | Yes* | Yes* |  |  |  |  |
|  | b) >60% subjects accounted for |  |  |  |  |  | Yes*:  94.4% | Yes* 93 % (asthma)  Yes* 84.3% (wheezing) |
|  | c) ≤ 60% subjects accounted for |  |  |  | Yes  29.1% | Yes  52.5% |  |  |
|  | d) no statement |  |  |  |  |  |  |  |

**Notes:** # see supplementary table 2 for ascertainment of exposure

**Supplementary table 5 (continue)**

|  | | Gehring U et al, 2015 [30] | | Ranzi A et al, 2014 [32] | | Aguilera I et al, 2013 [13] | | Fuertes E et al, 2013 [33] | | Gruzieva O et al, 2013 [34] Nordling E et al, 2008 [25] | Lindgren A et al, 2013 [35] | | | Clark NA et al, 2010 [36] | | Morgenstern V et al. 2007 [26] |
| --- | --- | --- | --- | --- | --- | --- | --- | --- | --- | --- | --- | --- | --- | --- | --- | --- |
| SELECTION | 1) Representativeness of the exposed cohort | | | | | | | | | | | | | | | |
|  | a) truly representative of the average in the community | Yes* | | Yes* | | Yes* | | Yes* | | Yes* | Yes* | | | Yes* | | Yes* |
|  | b) partially representative;  c) selected groups;  d) no description |  | |  | |  | |  | |  |  | | |  | |  |
|  | 2) Selection of the not exposed cohort | | | | | | | | | | | | | | | |
|  | a) drawn from the same community as the exposed cohort | Yes* | | Yes* | | Yes* | | Yes* | | Yes* | Yes* | | | Yes* | | Yes* |
|  | b) drawn from a different source;  c) no description |  | |  | |  | |  | |  |  | | |  | |  |
|  | 3) Ascertainment of exposure | | | | | | | | | | | | | | | |
|  | a) secure record  # | Yes* | | Yes* | | Yes* | | Yes* | | Yes* | Yes* | | | Yes* | | Yes* |
|  | b) structured interview;  c) written self-report;  d) no description |  | |  | |  | |  | |  |  | | |  | |  |
| SELECTION | 4) Demonstration that outcome of interest was not present at start of study | | | | | | | | | | | | | | | |
|  | a) yes |  | |  | |  | |  | |  |  | | |  | |  |
|  | b) no | Annual average exposure estimated at birth address and outcome (asthma or wheezing) from birth up to 14-16 years. | | Annual average exposure estimated at birth address and outcome (asthma) from birth up to 7 years. | | Annual average exposure estimated in the first year of life and outcome (wheezing) from birth up to 12-18 months | | Annual average exposure estimated at birth address and outcome (asthma) from birth up to 10 years | | Annual average exposure estimated in the first year of life and outcome from birth up to 12 years (asthma) and from birth up to 4 years (wheezing) | Annual average exposure estimated at birth address and outcome (asthma) from birth up to 1--6 years | | | Exposure estimated in the first year of life and outcome (asthma) from birth up to 36-59 months | | Average annual exposure estimated at birth address and outcome (wheezing) from birth up to 2 years |
| COMPARABILITY | 1) Comparability of cohorts on the basis of the design or analysis | | | | | | | | | | | | | | | |
|  | a) study controls for maternal smoking and history of asthma | Yes* | | Yes* | | Yes* | | Yes* | | Yes* | Yes* | | | Yes* | | Yes* |
|  | b) study controls for early life exposure and/or changes of residence | Yes*  (changes of residence only) | | Yes*  (changes of residence only) | | Yes*  (changes of residence only) | | Yes*  (changes of residence only) | | No | Yes*  (changes of residence only) | | | Yes* | | No |
| OUTCOME | 1) Assessment of outcome | | | | | | | | | | | | | | | |
|  | a) independent blind assessment |  | |  | |  | |  | |  |  | | |  | |  |
|  | b) record linkage |  | |  | |  | |  | |  | Yes* | | | Yes* | |  |
|  | c) self report | Parental reporting | | Parental reporting | | Parental reporting | | Parental reporting | | Parental reporting |  | | |  | | Parental reporting |
|  | d) no description |  | |  | |  | |  | |  |  | | |  | |  |
| OUTCOME | 2) Was follow-up long enough for outcomes to occur | | | | | | | | | | | | | | | |
|  | a) yes  (asthma diagnosis ≥ 6 years; wheezing ≥ 2 years) | Yes* | Yes* | |  | | Yes* | | Yes* | | |  |  | | Yes* | |
|  | b) no |  |  | | No | |  | |  | | | No | No | |  | |
|  | 3) Adequacy of follow up of cohorts | | | | | | | | | | | | | | | |
|  | a) complete follow up - all subjects accounted for at the last follow up |  | |  | |  | |  | |  | Yes* | | | Yes* | |  |
|  | b) >60% subjects accounted for | Yes* 98-79% | | Yes* 73.95% | | Yes* 83.2% | |  | | Yes* 82-91% |  | | |  | | Yes* 80.5% |
|  | c) ≤ 60% subjects accounted for |  | |  | |  | | Yes 55. 9% | |  |  | | |  | |  |
|  | d) no statement |  | |  | |  | |  | |  |  | | |  | |  |

**Notes:** # see supplementary table 2 for ascertainment of exposure
